# Anxiety and Depression during COVID-19 in Elite Rugby Players: The Role of Mindfulness Skills

**DOI:** 10.3390/ijerph182211940

**Published:** 2021-11-13

**Authors:** Kearnan Myall, Jesus Montero-Marin, Willem Kuyken

**Affiliations:** Department of Psychiatry, University of Oxford, Oxford OX3 7JX, UK; willem.kuyken@psych.ox.ac.uk

**Keywords:** anxiety, depression, athlete, elite sport, mindfulness, prospective study

## Abstract

The COVID-19 pandemic created stressors that raised the likelihood of elite athletes experiencing mental health problems. Understanding how individual traits promote resilience is key to offering treatments specific to this population. This prospective study explores the relationship between mindfulness skills, resilience, and athletic identity on anxiety and depression. The initial assessment was during the first UK lockdown April–May 2020 (T1), and the second during the return to competition July-August 2020 (T2). The sample was 160 elite rugby players. Measures included: Personal Health Questionnaire-9, General Anxiety Disorder-7, Cognitive Affective Mindfulness Scale, Connor-Davidson Resilience Scale, and Athletic Identity Measurement. The prevalence of anxiety and depression was profiled with descriptive statistics, and relationships between variables with bi-variate correlations and forward stepwise regression modelling. Depression decreased significantly between lock down (T1) and return to competition (T2) (*M*_T1_ = 4.20, *M*_T2_ = 3.24, *p* < 0.01), with no significant change in anxiety. Significant correlations were found between mindfulness, resilience, and anxiety and depression (≤0.001). Regression showed that mindfulness (T1) predicted lower anxiety and depression during the return to competition (T2) after controlling for baseline mental health symptoms. Returning to competition after lockdown was associated with a reduction in depression but not anxiety. Mindfulness skills potentially confer protection against anxiety and depression.

## 1. Introduction

Anxiety and depression represent a considerable health burden in the general population, and are the leading causes of mental health related diseases globally [1]. In elite athletes, anxiety and depression are also the most common mental health disorders, with a joint prevalence of 34% [2].

The COVID-19 pandemic has been associated with increased levels of psychological distress globally, although there appears to be significant demographic and geographic variability [3]. In the UK, during the first six months of the pandemic, the mental health of most adults remained close to pre-pandemic levels [4], but younger adults were found to be at greater risk of anxiety and depression [5]. Elite athletes are predominantly younger adults, and their careers overlap with the age range most commonly associated with the development of mental health disorders [6]. During times of increased stress, athletes have been shown to have higher rates of mental health disorders than the general population [7]. In elite rugby specifically, hyper-masculinity is a problematic feature of both the men’s and women’s game [8]. Hyper-masculinity is characterised by a negative attitude towards mental health, reduced help-seeking behaviour, and is associated with poor mental health [9]. Additionally, the exposure to risk of injury, a known contributor to poor mental health, is greater than most other sports [10]. This is especially true for head injuries, which are again widely associated with increased anxiety and depression [11]. In addition to these stressors, the COVID-19 pandemic has contributed to a real or perceived increased risk of infection, plus ongoing uncertainty, postponement, and cancelation of competitions [12]. There is a growing requirement to spend time in bio-secure bubbles away from regular sources of support, creating further unique stressors that expose athletes to a greater risk of mental health problems [13].

The treatment of anxiety and depression requires careful consideration in elite athletes. Pharmacological treatments may produce undesirable side effects that interfere with training, for example tricyclic and serotonin-norepinephrine reuptake inhibitor antidepressants require cardiac monitoring due to the frequency of high intensity exercise [14]. Psychological approaches are, therefore, recommended as a primary option [15]. Insight into personality traits that may confer protection against anxiety and depression are required to facilitate suitable support for the specific challenges athletes face [16]. Mindfulness is both a trait and a state, characterised by non-judgmental awareness and attention to present moment thoughts, emotions, and feelings [17]. Individuals with high levels of trait mindfulness have been shown to ruminate less [18], and have improved attentional control and emotional regulation compared to those with low trait mindfulness [19]. When focusing on elite athletes, these outcomes can benefit both mental health and athletic performance [20]. 

Mindfulness has been widely associated with resilience [21], with resilience thought to have a mediating role in the effect of mindfulness interventions on subjective wellbeing [22]. Resilience can be thought of as the capacity to tolerate and adapt to adverse life events [23]. It has been proposed that emotional resilience may be a mechanism of mindfulness, and resilience has been shown to partially mediate the effect of mindfulness and self-compassion on depression [24]. However, the role of mindfulness and resilience in supporting mental health in elite sport is not well understood. Characteristics such as athletic identity, which encompass how an individual’s beliefs, experiences, and generalisations about oneself rely on being an athlete [25], add a more complex dimension to the relationship between mindfulness and resilience. Athletes with higher levels of mindfulness are thought to have lower athletic identity [20], but it is not clear whether this benefits their mental health. Studies investigating the effect of high athletic identity have observed various outcomes ranging from increased motivation and reduced burnout [26], to high levels of rumination and catastrophising [27].

To understand the effect trait mindfulness, resilience, and athletic identity had on mental health during the overarching stressor of the COVID-19 pandemic, including the first lockdown and the return to training and competition, an investigation into the mental health of elite rugby players was undertaken. To the best of the authors’ knowledge, research into the mental health of elite rugby players during the COVID-19 pandemic has not been published. In this context, the aims of this study were: (1) to describe the prevalence of symptoms of anxiety and depression in elite rugby players during COVID-19 lockdown and when returning to competition; (2) to test whether levels of trait mindfulness, resilience, and athletic identity were associated with anxiety and depressive symptomatology during lockdown; and (3) to evaluate if levels of trait mindfulness, resilience, and athletic identity during lockdown predict symptoms of anxiety and depression after lockdown when returning to competition. 

## 2. Materials and Methods

### 2.1. Participants and Procedure

Eligibility criteria required that participants were: (a) current elite rugby players, classified as competing internationally or in top-tier professional leagues, to accord with a widely used criteria used to define elite athletes [28]; and (b) over the age of 18. In total, 160 professional rugby players were included in the full analysis of the study. The sample consisted of 151 male (94.4%) and 9 female (5.6%) rugby players. The sample is, therefore, slightly weighted toward male players based on an estimated 1100 male [29] and 230 female international/professional players [28]. Overall, this sample accounted for roughly 12% of all elite rugby players in the UK and Ireland. See Table 1 for participant characteristics. A single group linear regression power calculation was conducted. An expected standardised slope coefficient of −0.10 for the regression of mindfulness as a predictor of anxiety/depressive symptoms, using a significance level α = 0.05, and a power (1 − β) of 0.90, resulted in a minimum sample size *n* = 96.

A prospective two-wave study was designed to measure symptoms of anxiety and depression during the first COVID-19 lockdown-(T1), and during the return to competition-(T2). Elite rugby players from the UK and Ireland were approached to take part in the study in accordance with The University of Oxford Medical Sciences Interdivisional Research Ethics Committee (reference: R69230/RE001). Recruitment was carried out via The Rugby Players Association in England, after approval from the Rugby Football Union’s board of medicine. Additionally, individuals from professional teams were asked to participate in the study via coaches and medical staff. An invitation to participate in the study was sent via email to prospective participants during T1, between 14th April 2020 and 10th May 2020. This period of time aligned with the shutting down of non-essential activities, restrictions on movement, and bans on gatherings of groups of people in Europe and North America. The invitation contained an electronic link to Jisc online surveys (link; accessed date: Day, Month, Year)and opened on participant information. Once participants gave informed consent, they were asked to complete the survey. The survey contained questions from four widely used questionnaires (described below), plus five questions capturing: country, age, sex, injury status, and email address. Participants were then asked for consent to be contacted for the follow up. Follow up responses were gathered between 9th July and 11th August 2020-(T2). Reminders were sent via the Rugby Players Association and representatives from individual clubs. This period of time was chosen to align with the return to training for athletes in Europe. The follow-up timepoint measured anxiety, depression, and fitness status only.

### 2.2. Instruments

Trait mindfulness, resilience, and athletic identity were measured at T1 to evaluate the associations between them and anxiety and depressive symptoms at T1 and T2. Socio-demographic information including age, sex, country, and fitness status was measured using standard questions at the start of the survey.

#### 2.2.1. Anxiety

General Anxiety Disorder-7 Questionnaire (GAD7). This measure has adequate properties for detecting anxiety [30], and it has been validated in elite athletes [31]. Participants were asked: “over the last two weeks how often have you been bothered by the following problems?”, e.g., “trouble relaxing”. Responses range from 0 (“not at all”) to 4 “(nearly every day”) on a four-point Likert-type scale. The sum of scores for the seven questions indicate the severity of anxiety symptoms on a continuous scale from 0 (no symptoms) to 21 (severe anxiety). The following cut-off points are widely used: ≥5 = mild, ≥10 = moderate anxiety, ≥15 = moderately severe, and ≥20 = severe. Scores of ≥10 are treated as clinically relevant [32]. Internal consistency (Cronbach’s *α*) in the present study was *α* = 0.83 at T1 and *α* = 0.88 at T2. 

#### 2.2.2. Depression

Personal Health Questionnaire-9 (PHQ9). This measure has good properties for detecting symptoms of depression [30], and it has been validated in elite athletes [31]. Participants were asked: “over the last two weeks how often have you been bothered by the following problems?*”*, e.g., “feeling down, depressed, or hopeless”. Responses range from 0 (“not at all”) to 4 (“nearly every day”) on a four-point Likert-type scale. The sum of scores for the nine questions indicate the severity of symptoms on a continuous scale from 0 (no symptoms) to 27 (severe depression). The following cut-off points are widely used: ≥5 = mild, ≥10 = moderate, ≥15 = moderately severe, and ≥20 = severe. Scores of ≥10 are treated as clinically relevant [33]. Internal consistency in the present study was *α* = 0.82 at T1 and *α* = 0.84 at T2.

#### 2.2.3. Trait Mindfulness

Cognitive and Affective Mindfulness Scale–Revised (CAMS-R). We assessed trait mindfulness with the 12-item CAMS-R questionnaire [34], which includes use in athlete populations [35]. Participants were asked to rate how much a statement like “I am easily distracted,” applies to them. Answers range from “Rarely or not at all” to “Always” and are scored on a four-point Likert-type scale. Items 2, 6, and 7 are reversed scored and are summed to give a total score from 12–48. Higher scores reflect greater trait mindfulness. Internal consistency in the present study at T1 was *α* = 0.80.

#### 2.2.4. Resilience

Connor Davidson Resilience Scale-10 (CD-RISC). Resilience was measured by the CD-RISC, which has good psychometric properties [23], and has been validated in elite athletes showing support for the convergent validity and unidimensional factorial structure of the scale [36]. Participants were asked how true a statement like: “coping with stress can strengthen me” is for them. Responses range from 0 (“Not true at all”) to 4 (“True nearly all the time”), and are scored on a four-point Likert-type scale. Scores are summed on a continuous scale of 0–40 with higher scores being indicative of greater resilience. Internal consistency in this study at T1 was *α* = 0.86.

#### 2.2.5. Athletic Identity

Athletic Identity Measurement Scale (AIMS). To measure athletic identity, we used the AIMS, a questionnaire that was designed for use in athletes and has acceptable psychometric properties [37]. Participants rate their agreement with statements, such as: “I consider myself an athlete,” on a seven-point Likert-type scale ranging from 1 (“strongly disagree”) to 7 (“strongly agree”). Scores are summed on a continuous scale of 7–70, with higher scores indicative of greater athletic identity. Internal consistency in the present study at T1 was *α* = 0.80.

### 2.3. Statistical Analysis

Missing values were very low (0.01%), and were imputed using the mean of the total score for the respective questionnaire. To understand the prevalence of symptoms of anxiety and depression at the two different stages of the beginning of the COVID-19 pandemic, we described the percentage of individuals who scored: ≥5 (mild), ≥10 (moderate), ≥15 (moderately severe), and ≥20 (severe) on the GAD7 and PHQ9 [32,33]. Chi-squared tests were used to evaluate possible differences in the proportions of individuals at each cut-off point between T1 and T2. The mean difference of sample scores for anxiety and depression between T1 and T2, and in terms of the country, were also calculated using dependent t-tests, and ANOVA was used to explore the differences in means for sociodemographic variables with multiple categories. To test if symptoms of anxiety and depression were associated with trait mindfulness, resilience, and athletic identity, correlation coefficients were calculated using Spearman’s coefficients. To evaluate if these individual traits at T1 might predict symptoms of anxiety and depression at T2, a forward stepwise regression analysis was conducted to produce two models (e.g., one for anxiety, another for depression), which included mindfulness, resilience, and athletic identity as independent variables, as well as the demographic variables age, sex, country, and fitness status. Anxiety and depression scores at T1 were also included in the T2 regression models to account for baseline symptoms. This method removes the portion of the T2 score that is linearly predicted by T1 score [38].

The RStudio-Version 1.2.5019 statistical package was used for analyses. The statistical significance was set at *p* < 0.05 on both sides.

## 3. Results

The e-questionnaire was sent to 520 elite rugby players. At T1, 191 (36.7%) initial responses were received, and at T2 160 athletes (83.8% of initial responders) completed the follow up and were included in the full analysis. There were no significant differences between the T1 GAD7 and PHQ9 scores of participants who completed the follow-up and those who did not. Participant characteristics are available in Table 1.

### 3.1. Anxiety and Depressive Symptoms

Mild symptoms of depression (PHQ9 ≥5) were significantly more prevalent at T1 (36.25%) than at T2 (20.63%) (8.85, *p* < 0.01). There were no significant differences in the prevalence of moderate (PHQ9 ≥ 10) or moderately severe (PHQ9 ≥ 15) depression between T1 and T2, although the number of athletes reporting symptoms in each brackets reduced by half (Table 1). The mean depression score at T1 (*M* = 4.20, *SD* = 3.59) was significantly greater (*p* = 0.02) than at T2 (*M* = 2.97, *SD* = 3.24). The prevalence of anxiety between T1 and T2 did not differ significantly at any of the cut-off points, nor did the mean difference between the two time points (Table 1). At both T1 and T2, 5% of rugby players (8/160) reported experiencing thoughts about suicide or self-harm in the past two weeks, as measured by item nine on the PHQ9 questionnaire.

By nation, players in England (*n =* 135/160 − 84%) and Ireland (*n* = 23/160 − 14%) made up the majority of this sample. Comparing the two subgroups, there were no differences in the mean anxiety or depression scores at T1. At T2, however, players in England reported significantly higher mean scores than players in Ireland for anxiety (*M*_1_ = 4.2, *SD*_1_ = 3.7, *M*_2_ = 2.2, *SD*_2_ = 2.1, *p* = 0.02), and depression (*M*_1_ = 3.2, *SD*_1_ = 3.4, *M*_2_ = 1.7, *SD*_2_ = 1.8, *p* = 0.03). There were no differences in the mean scores for anxiety and depression at T1 or T2 between fit and injured players, or between age groups.

### 3.2. Bivariate Analysis

As expected, mindfulness showed a moderate inverse correlation with anxiety and depression at both T1 and T2. Resilience showed a similar moderate inverse correlation with anxiety and depression at T1, although this weakened with depression at T2. Athletic identity presented a moderate inverse correlation with mindfulness at T1, but it showed only a weak positive association with anxiety and depression at T1 and T2. Full details in Table 2.

### 3.3. Predictors of Anxiety and Depression at T2

As can be seen in Table 3, Model 1 (depression) indicated that five variables explained 37.2% of the variance (F(8,151) = 12.8, *p* < 0.001). T1 depression acted as a control for baseline symptoms, and mindfulness significantly predicted a small amount of protection against depressive symptoms (*B* = −0.10, *p =* 0.03). Players based in Ireland were predicted to have lower depression (*B* = −1.50, *p* = 0.01), as were those aged 28–32 (*B* −1.25, *p* = 0.03) Sex was also included in the final model. Being a male athlete predicted lower depression (*B* = −1.47, *p* = 0.10), but this was not statistically significant, possibly due to the small number of female athletes in the sample. As can be seen in Table 3, Model 2 (anxiety) showed that five variables explained 32% of the variance (F(6,153) = 13.49, *p* < 0.001). T1 anxiety acted as a control for baseline symptoms. Being injured at the start of the first lockdown (T1) predicted significantly higher anxiety at T2 (*B* = 1.27, *p* = 0.02). Mindfulness significantly predicted a small amount of protection against anxiety (*B* = −0.13, *p* = 0.01). Similarly to depression, players based in Ireland were predicted to have lower anxiety (*B* = −1.59, *p* = 0.02), and sex was again also included in the final model despite not being statistically significant (*B* −1.81, *p* = 0.08), likely due to the small number of female athletes in the sample.

## 4. Discussion

This study aimed to describe the prevalence of symptoms of anxiety and depression, and their relationships with trait mindfulness, resilience, and athletic identity in elite rugby players during the first COVID-19 lockdown and the return to competition.

Addressing the first aim of the study, our results showed that, in general, symptoms of anxiety and depression were lower in elite rugby players than the general population over this time period, and considerably lower than those aged 18–34 (29.6% anxiety, 31.5% depression), the group most similar by age to the study sample [5]. This aligns with the findings from other research that reported elite athletes had lower stress and higher biopsychosocial functioning scores than novice athletes during the early stages of the pandemic [39], and that professional athletes who were forced to self-isolate had better mental health status than non-athletes [40].

The mean score for depression significantly reduced between lockdown-(T1) and the return to competition-(T2), suggesting that the return to sport after lockdown had a beneficial impact on mental health. In support of this, the proportion of rugby players who experienced mild symptoms of depression significantly reduced between lockdown and the return to competition, from 36% to 21%. Although not statistically significant, the proportion of rugby players who experienced moderate or moderate-severe symptoms of depression halved between lockdown and the return to competition, from 7.5% to 3.8%. Anxiety, however, did not differ significantly at any level between lockdown and the return to competition. One explanation is that the reduction in depression from lockdown to the return to competition is likely to be due to the fact that returning to training and competition increased motivation, self-worth, and energy levels, particularly in those with only mild symptoms of depression. On the other hand, fear of infection, worry about the health and wellbeing of family and friends, and concerns about fitness levels and missed training opportunities are likely to have contributed to anxiety levels remaining similar between the two timepoints [12]. Sub-group analysis found that the mean anxiety and depression scores of players in Ireland was significantly lower than those in England during the return to competition, but not during the first lockdown. This finding must be interpreted with caution, as 22 players in Ireland were sampled from a single club, so it is not possible to determine whether the environment of the country or the club itself influenced the more significant reduction in symptoms. Individuals reporting suicidal thoughts remained consistent at 5% across both timepoints. Further research is required to interpret this finding.

The second aim of the study was to test the associations between trait mindfulness, resilience, athletic identity, and anxiety and depression. Anxiety and depression showed moderate inverse associations with mindfulness and resilience, respectively. The relationship between athletic identity and anxiety, and athletic identity and depression, were both weak and non-significant, suggesting that athletes with high athletic identity were not negatively affected by the forced absence from training and competition any more than those with lower athletic identity. Forced absences from sport, such as injury or premature retirement, are known to negatively affect an athlete’s wellbeing and personal identity [41], but it has been reported that the absence due to the COVID-19 pandemic may have imbedded deeper values such as purpose, and a recognition of the value that elite sport holds in society [42], which might have had a protective effect.

The third aim of the study was to evaluate if levels of trait mindfulness and resilience, as well as athletic identity predict symptoms of anxiety and depression after lockdown when returning to competition. Controlling for baseline depression at lockdown, mindfulness, country, age, and sex were found to be significant predictors of depression during the return to competition. Sex was a non-significant predictor, likely to be due to the small number of female athletes, but remained in the model as this finding aligns with a wealth of evidence suggesting female athletes report higher levels of depression and anxiety than male athletes [2]. Being based in Ireland also appeared to afford some protection against depression, but as previously reported it is not possible to infer if this benefit came from the environment of the country or the rugby club that the majority of the sample was taken from. Players aged 28–32 were also afforded protection against depression compared to younger athletes, in line with findings from the general population that younger adults had worse mental health outcomes during the pandemic [43]. This protection was not afforded to the oldest age group in the sample (≥33+), possibly due to the prospect of losing the final years of their athletic career to the pandemic. Similarly, controlling for baseline anxiety at lockdown, mindfulness, sex, country, and injury status were found to be predictors of anxiety during the return to competition. Again sex was non-significant but remained in the model. The inclusion of injury at lockdown-(T1) as a predictor for higher anxiety at the return to competition-(T2) is an interesting finding in light of the fact that depression, but not anxiety, reduced between lockdown and the return to competition. Our interpretation of this finding is that injured athletes missed out on training, and physiotherapy/medical care during lockdown. This is likely to have increased anxiety during the return to competition if their perceived level of physical fitness was not at the desired standard. As expected, and in line with previous research, mindfulness was found to predict future symptoms of anxiety and depression [19]. Athletes who had higher levels of trait mindfulness during lockdown were likely to have lower scores for anxiety and depression after lockdown. The benefits to mental health from higher trait mindfulness are thought to be delivered through effective emotional regulation, self-compassion, acceptance, and the ability to identify negative thought patterns and reduce rumination [19].

The association between resilience and mindfulness is well documented [21] and this study extends the literature by showing the same relationship in elite athletes. Resilience, however, did not significantly add explained variance in the prediction of anxiety or depression in the regression analysis, and the strength of the association with depression reduced from lockdown to the return to competition. This suggests that resilience may be a less stable trait than mindfulness and the results from this study support previous findings that an individual’s resilience fluctuates based on affective temperament [44]. Depressed individuals will exhibit lower resilience but, with treatment of symptoms, resilience can be improved [23].

The main limitation of our prospective study design is that it is only possible to suggest possible but not causal relationships, and measurements between variables were taken at just two time points. Future randomised controlled trials will have to test the potential protective role of mindfulness skills. The method of availability sampling may also be considered a limitation as there is no guarantee the sample will be representative of all elite rugby players in the UK. Those with particularly good or poor mental health may have been more or less inclined to take part in the study, and the sample was slightly weighted towards male players. There are also many variables that it was not feasible to control for, considering the difficult period of time that the data was collected in, such as history of mental health disorders and current treatment, or direct impact of COVID-19 pandemic (i.e., bereavement, loss of income, etc.).

The high standard of professional and/or international athletes included in the sample is, however, a strength, and whilst we acknowledge that this group may not be representative of all elite athletes, the data was collected during a difficult period of time under a general stressor that is of high interest. Future research should focus on further understanding the individual variances that influence mental health within the context of elite sport, and the relationship between resilience and mindfulness skills, to understand the mechanisms by which they combine to provide protection from anxiety and depression.

## 5. Conclusions

Symptoms of anxiety and depression in elite rugby players were lower than those in members of the public of a similar age at the start of the COVID-19 pandemic. Depression reduced from the period of lockdown to the return to competition but anxiety did not. Higher levels of mindfulness skills might confer protection against anxiety and depression in this population.

## Figures and Tables

**Table 1:** Sample Characteristics.

	*n* (T1)	% (T1)	*n* (T2)	% (T2)	Prop Diff (*p*)	Mean (T1)	SD (T1)	Mean (T2)	SD (T2)	Mean Diff (*p*)
Country										
England	135	84.38	135	84.38						
Ireland	23	14.38	23	14.38						
Scotland	2	1.25	2	1.25						
Sex										
Male	151	94.38	151	94.38						
Female	9	5.63	9	5.63						
Age										
18–22	39	24.38	39	24.38						
23–27	59	36.38	59	36.38						
28–32	53	33.13	53	33.13						
≥33	9	5.63	9	5.63						
Fitness status										
Fit	122	76.25	147	91.88						
Injured	38	23.75	13	8.12						
Anxiety	160	160	160	160		4.43	3.52	3.87	3.61	0.16
Mild	63	39.38	51	31.88	0.19					
Moderate	15	9.34	12	7.5	0.68					
Mod-Severe	2	1.25	2	1.25	1					
Depression	160	160	160	160		4.2	3.59	2.97	3.24	<0.01
Mild	58	36.25	33	20.63	<0.01					
Moderate	12	7.5	6	3.75	0.23					
Mod-Severe	4	2.5	2	1.25	0.68					
Mindfulness	160	160	160	160		32.16	5.1			
Resilience	160	160	160	160		29.48	5.22			
Athletic ID	160	160	160	160		48.77	8.28			

Anxiety was measured by the General Anxiety Disorder 7 questionnaire. Depression was measured by the Patient Health Questionnaire 9. Cut-off points: Mild = ≥5, Moderate ≥ 10, Moderately-Severe = ≥15. Prop diff = Proportional difference. SD = Standard deviation. T1 = First UK lockdown due to COVID-19 (April-May 2020). T2 = The return to competition (July-August 2020).

**Table 2:** Correlation coefficients.

	Anxiety (T1)	Depression (T1)	Anxiety (T2)	Depression (T2)	Resilience	Mind	Athletic ID
AnxietyT1	-	<0.001	<0.001	<0.001	<0.001	<0.001	>0.05
DepressionT1	0.50	-	<0.001	<0.001	<0.001	<0.001	0.17
AnxietyT2	0.55	0.45	-	<0.001	<0.001	<0.001	0.08
DepressionT2	0.40	0.55	0.65	-	<0.001	<0.001	0.09
Resilience	−0.32	−0.32	−0.35	−0.22	-	<0.001	0.72
Mindfulness	−0.38	−0.35	−0.41	−0.38	0.58	-	<0.01
Athletic ID	0.15	0.11	0.14	0.14	0.03	−0.32	-

Correlation coefficients between measured variables (below) and their corresponding *p*-values (above). Mind = Mindfulness. Athletic ID = Athletic identity. T1 = First UK lockdown due to COVID-19 (April-May 2020). T2 = The return to competition (July-August 2020).

**Table 3:** Linear Regressions.

Model 1: Stepwise Regression Model for Depression (PHQ9) at T2						
	Adj*R^2^*	*F*-Statistic	*B*	*β*	*SE*	*p*
	0.37	12.8				<0.001
T1 Depression			0.44	0.50	0.06	<0.001
T1 Mindfulness			−0.10	−0.14	0.04	0.03
Country (Ireland)			−1.50		0.59	0.01
Age (28–32)			−1.25		0.55	0.03
Sex (M)			−1.47		0.89	0.10
Model 2: Stepwise Regression Model for Anxiety (GAD7) at T2						
	Adj-$R^{2}$	*F*-Statistic	*B*	*β*	*SE*	*p*
	0.32	13.49				<0.001
T1 Anxiety			0.44	0.44	0.07	<0.001
T1 Fitness (Injured)			1.27		0.56	0.02
T1 Mindfulness			−0.13	−0.17	0.05	0.01
Country (Ireland)			−1.59		0.67	0.02
Sex (M)			−1.81		1.03	0.08

Depression measured by Patient Health Questionnaire 9. Anxiety measured by the Generalised Anxiety Disorder 7 questionnaire. Adj*R^2^* = Adjusted R-squared value. *B* = non-standardised slope. *β* = standardised slope. *SE* = standard error. (M) = Male. T1 = First UK lockdown due to COVID-19 (April-May 2020). T2 = The return to competition (July-August 2020).

## Data Availability

The data and code that support the findings of this study are openly available at The Bodleian Libraries, Oxford University Research Archive (ORA) at: https://ora.ox.ac.uk/objects/uuid:984230bc-7e7-4a22-9b32-ad59340f1ce7 (accessed on 9 November 2021, https://doi.org/10.5287/bodleian:qqOGKXjXQ) (accessed on 9 November 2021).
